# Col1A1 Production and Apoptotic Resistance in TGF-β1-Induced Epithelial-to-Mesenchymal Transition-Like Phenotype of 603B Cells

**DOI:** 10.1371/journal.pone.0051371

**Published:** 2012-12-07

**Authors:** Jun Liu, Alex N. Eischeid, Xian-Ming Chen

**Affiliations:** Department of Medical Microbiology and Immunology, Creighton University School of Medicine, Omaha, Nebraska, United States of America; IISER-TVM, India

## Abstract

Recent studies have suggested that proliferating cholangiocytes have an important role in the induction of fibrosis, either directly via epithelial-to-mesenchymal transition (EMT), or indirectly via activation of other liver cell types. Transforming growth factor beta 1 (TGF-β1), a critical fibrotic cytokine for hepatic fibrosis, is a potent EMT inducer. This study aimed to clarify the potential contributions of TGF-β1-induced EMT-like cholangiocyte phenotype to collagen production and cell survival of cholangiocytes *in vitro*. Mouse cholangiocytes (603B cells) were treated with TGF-β1 and EMT-like phenotype alterations were monitored by morphological changes and expression of EMT-associated genes. Alterations in Col1A1 gene, Col1A1-associated miR-29s, and pro-apoptotic genes were measured in TGF-β1-treated 603B cells. Snail1 knockdown was achieved using shRNA to evaluate the contribution of EMT-associated changes to Col1A1 production and cell survival. We found TGF-β1 treatment induced partial EMT-like phenotype transition in 603B cells in a Snail1-dependent manner. TGF-β1 also stimulated collagen α1(I) expression in 603B cells. However, this induction was not parallel to the EMT-like alterations and independent of Snail1 or miR-29 expression. Cells undergoing EMT-like changes showed a modest down-regulation of multiple pro-apoptotic genes and displayed resistance to TNF-α-induced apoptosis. TGF-β1-induced apoptosis resistance was attenuated in Snail1 knockdown 603B cells. TGF-β1-induced Col1A1 production seems to be independent of EMT-like transition and miR-29 expression. Nevertheless, TGF-β1-induced EMT may contribute to the increased survival capacity of cholangiocytes via modulating the expression of pro-apoptotic genes.

## Introduction

Liver fibrosis results from the accumulation of interstitial or “scar” extracellular matrix (ECM) after either acute or chronic liver injury. The advanced stage of hepatic fibrosis is liver cirrhosis, which gradually destroys the hepatic architecture and causes significant deaths worldwide. Mechanistic studies have been focused on hepatic stellate cells (HSCs), as these cells can undergo “activation” into proliferative and fibrogenic myofibroblast-like cells during liver injury [1], [2]. Other live cell types may also play an important role in the pathogenesis of liver fibrosis [3]. Pathologically, ECM proteins are predominantly deposited around the portal region during development of hepatic fibrosis [4]. Cholangiocytes, epithelial cells lining the biliary tree, become aberrantly accumulated in the portal region and form clustered bile ducts during hepatic fibrosis [5]. Such reaction reflects a dysregulated balance between cell growth and cell death, referred as “ductular reaction” [6]. Recent studies demonstrate that cholangiocytes display features of epithelial-to-mesenchymal transition (EMT) during hepatic fibrosis [4], [7]–[9]. EMT describes the molecular reprogramming and phenotypic changes involved in the conversion of polarized immotile epithelial cells to motile mesenchymal cells [10]. This process allows the remodeling of tissues during embryonic development and is recently implicated in tumor progression and development of fibrosis [11], [12]. Pathophysiologically, cholangiocytes with mesenchymal features may contribute to the generation of fibroblast-like cells to facilitate collagen production and promote the “ductular reaction” at the portal region during hepatic fibrosis [13], [14].

Transforming growth factor beta (TGF-β) is the prototype of most powerful EMT inducer in many epithelial tissues [15]. Interestingly, TGF-β1 is a critical fibrotic cytokine which plays an important role in the initiation and progression of liver fibrosis [16]. TGF-β1 has been demonstrated to induce collagen production as well as EMT-associated changes in several liver cell types, including cholangiocytes *in vitro* and *in vivo* [8], [17]–[19]. Uncovering unique EMT-associated molecular events in cholangiocytes in response to TGF-β1 may identify valuable targets for anti-fibrotic therapies. Nevertheless, it’s not clear whether TGF-β1-induced collagen production in cholangiocytes depends on the occurrence of EMT. Whether TGF-β1 promotes “ductular reaction” through induction of cholangiocyte EMT is also still obscure.

EMT can be viewed as a manifestation of extreme epithelial cell plasticity, characterized by loss of polarity, loss of epithelial markers, cytoskeletal reorganization, and transition to a spindle-shaped morphology concomitant with acquisition of mesenchymal markers [10]. Loss of epithelial adhesion does not necessarily lead to full transition to a mesenchymal phenotype [20]. EMT in response to TGF-β1 is mediated by the activation of a variety of downstream mediators of TGF-β1 signaling, including Smads and Snails [15]. Recent studies indicate that epithelial cells undergoing EMT also display distinct expression profile of microRNAs (miRNAs), a class of small regulatory RNAs which suppress gene expression at post-transcriptional level by base pairing with the 3′ untranslated regions (UTRs) of mRNAs [21]. Given the importance of miRNAs in gene regulation, they may play a pathogenic role in the many aspects of EMT, such as collagen production. Indeed, miR-29s have been demonstrated to directly target 3′UTRs of collagen mRNAs and downregulation of miR-29 family has been shown to correlate with the development of fibrosis in several tissues [22]–[24]. Although the detailed mechanisms for miR-29 downregulation during fibrosis remain to be elucidated, TGF-β1 decreases the transcription of this miRNA family in myofibroblasts *in vitro* [24]. In addition, EMT is well established to be associated with death resistance [10]. The main inducers of EMT can also promote apoptosis, suggesting that EMT could potentially serve as a pathway for escape from cell death depending on the particular cytokine milieu [25], [26]. It has recently been found that Snail1 directly regulates the expression of apoptosis genes [27], suggesting that down-regulation pro-apoptotic gene by EMT-associated transcriptional factor may contribute to the development of ductular reaction during fibrosis.

Using a non-tumorigenic mouse 603B cholangiocyte cell line, in this study, we investigated the development of EMT induced by TGF-β1, its relationship to collagen production and finally its involvement with the acquisition of apoptotic resistance in cholangiocytes *in vitro*. The data we report here show that TGF-β1 induces EMT-like phenotypic alterations and promotes collagen α1(I) (Col1A1) production in 603B cells. However, TGF-β1-induced Col1A1 production is independent of EMT-like alterations and miR-29 expression. Moreover, TGF-β1-induced EMT may contribute to the increased survival capacity of cholangiocytes via modulating the expression of pro-apoptotic genes.

**Figure 1:**
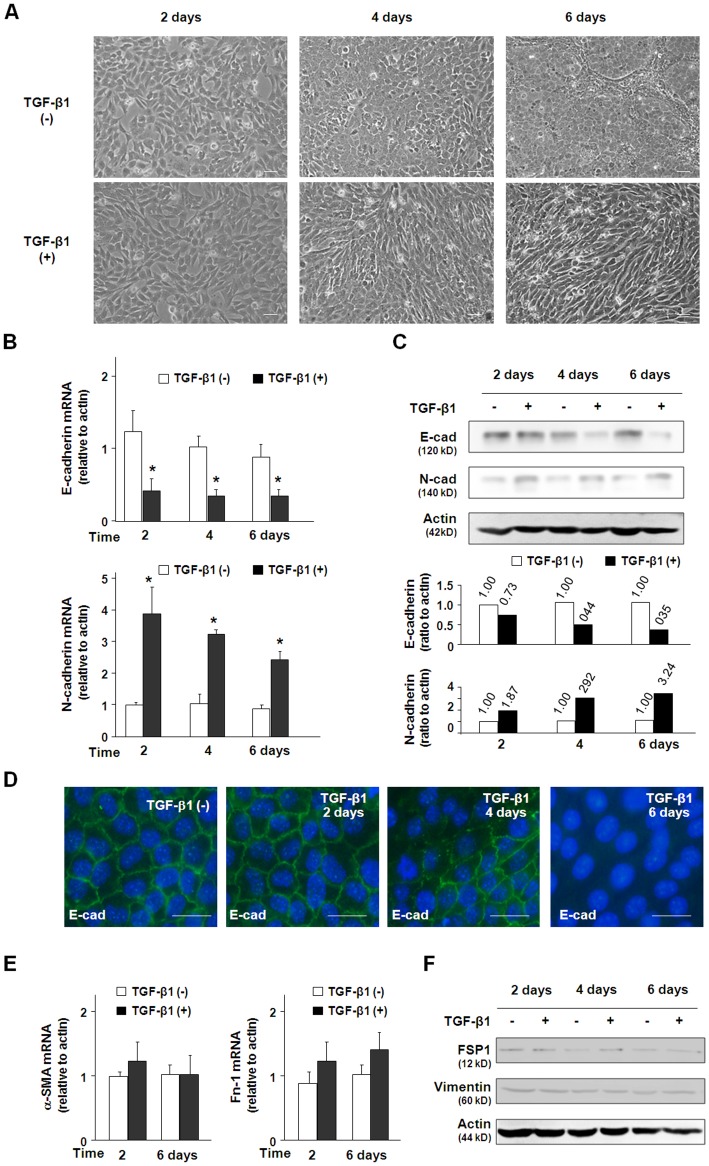
TGF-β1 induces EMT-associated changes in 603B cells. (A) Morphological alterations of 603B cells induced by TGF-β1 (3 ng/ml) at indicated time points. Upper panel are representative phase images showing the 603B cells cultured in the absence of TGF-β1 and lower panel shows the 603B cells stimulated with TGF-β1. After TGF-β1 exposure, 603B cells gradually assumed a spindle-like shape. (B) Alterations of E-cadherin and N-cadherin mRNA expression in 603B cells after exposure to TGF-β1 for various periods of time as assessed by qRT-PCR. TGF-β1 treatment induced steady down-regulation of E-cadherin and up-regulation of N-cadherin. The qRT-PCR results shown represent an average of three independent experiments. (C) Cellular levels of E-cadherin and N-cadherin proteins in TGF-β1-treated 603B cells as determined by Western blot. Consistent with qRT-PCR analysis, TGF-β1 induced down-regulation of E-cadherin and up-regulation of N-cadherin, respectively. Representative blots from three independent experiments are shown and actin was blotted to ensure equal loading. Densitometric levels of E-cadherin and N-cadherin signals were quantified and expressed as the ratio to actin. (D) Decreased cell membrane distribution of E-cadherin protein in 603B cells in respond to TGF-β1 stimulation as assessed by immunofluorescent staining. E-cadherin was stained green and DAPI stained nuclei blue. (E) α-SMA and Fn-1 mRNA expression levels were determined by qRT-PCR and (F) FSP-1 and vimentin protein expression levels were determined by Western blot. No significant change of these genes was detected. Values are means ± SE. *p<0.05 compared to non-TGF-β1-treated cells; E-cad = E-cadherin; N-cad = N-cadherin. Bar = 10 µM.

## Materials and Methods

### Cell Culture and TGF-β1 Treatment

A non-tumorigenic mouse cholangiocyte cell line 603B, was maintained in Dulbecco's modified Eagle's medium (Gibco) with 10% fetal bovine serum (Invitrogen). These cells were developed by transfection with the SV40 T antigen and they display typical morphology of well-differentiated cholangiocytes, express biliary epithelial cell markers consistent with biliary function and with the tight junctions between cells as previously documented [28]. 603B cells were seeded at 5% confluence and, 18 h later, were moved to a medium with 5% fetal bovine serum. TGF-β1 (R&D System) was added to a finial concentration of 3 ng/mL. Medium was changed every other day.

**Figure 2:**
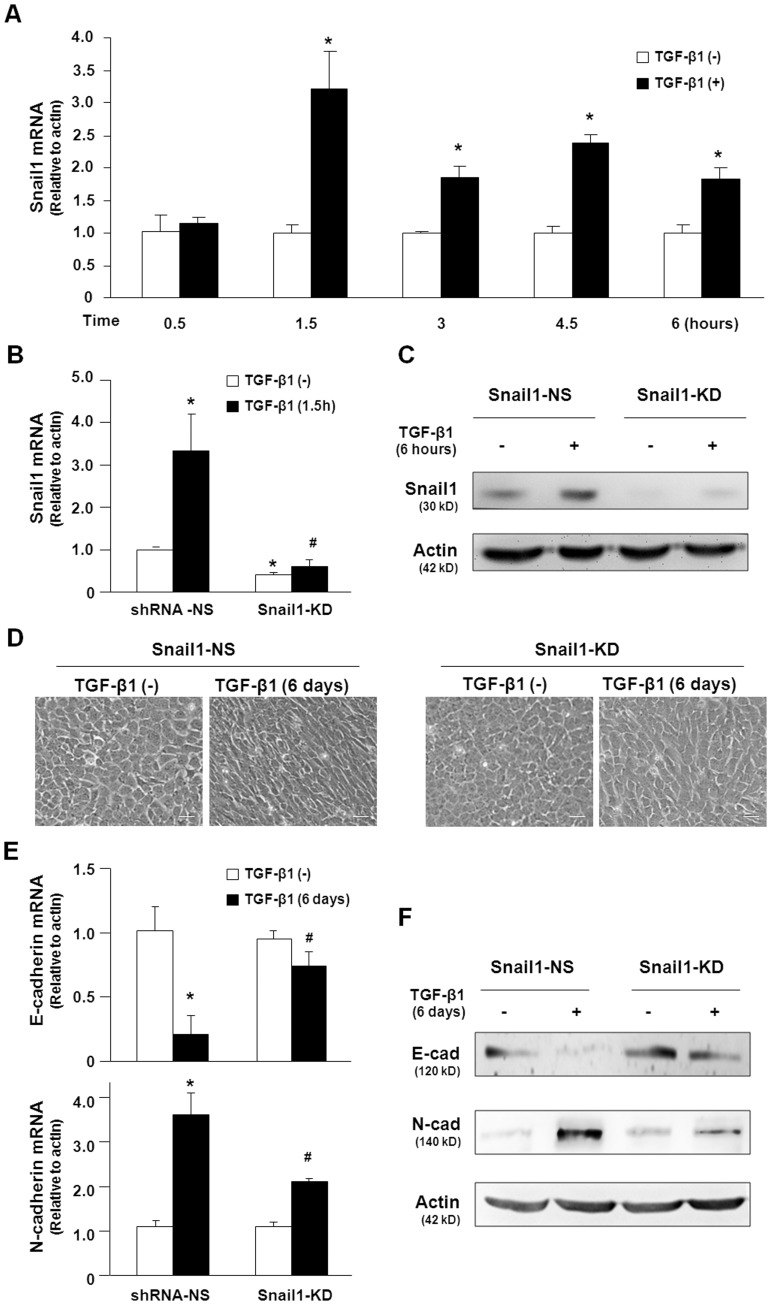
Snail1 knockdown attenuates TGF-β1-induced EMT-associated changes in 603B cells. (A) qRT-PCR revealed the rapid up-regulation of Snail1 mRNA levels in 603B cells after TGF-β1 (3 ng/ml) treatment. Data represent an average of three independent experiments. (B) Snail1 shRNA but not control shRNA abolished the upregulation of Snail1 mRNA levels by TGF-β1 in 603B cells. Cells stably expressing the Snail1 shRNA or control shRNA were exposed to TGF-β1 (3 ng/ml) for 1.5 h and expression of Snail1 mRNA levels were determined by qRT-PCR. (C) Snail1 shRNA decreases Snail1 protein expression in 603B cells. Cells stably expressing the Snail1 shRNA or control shRNA were exposed to TGF-β1 (3 ng/ml) for 6 h and expression of Snail1 protein levels were determined by Western blot. (D) Morphological changes of 603B cells stably expressing control shRNA or Snail1 shRNA after TGF-β1 (3 ng/ml) treatment for 6 days. (E) and (F) Effects of Snail1 knockdown on E-cadherin and N-cadherin expression in 603B cells following TGF-β1 treatment. Cells stably expressing the control shRNA or Snail1 shRNA were treated with TGF-β1 (3 g/ml) for 6 days and expression of E-cadherin and N-cadherin were determined by qRT-PCR (E) and Western blot (F), respectively. Values are means ± SE. *p<0.05 compared to non-TGF-β1-treated cells; ^#^ *p*<0.05 compared to cells expressing the shRNA-NS after TGF-β1 treatment; E-cad = E-cadherin; N-cad = N-cadherin; shRNA-NS = non specific shRNA control; Snail1-KD = snail shRNA knockdown. Bar = 10 µM.

### Establishment of Cells Stably Expressing Snail1 shRNA

Cells were transfected with control shRNA or shRNA against murine Snail1 (Origene) with lipofectamin2000. Transfection efficiency was 40% with this method. Forty-eight hours post-transfection, cells were selected with puromycin (1 µg/mL) for 3 weeks. Drug-resistant clones were isolated, expanded and confirmed by PCR and Western blot. All experiments were conducted using pools of colonies to avoid a clonal bias.

**Figure 3:**
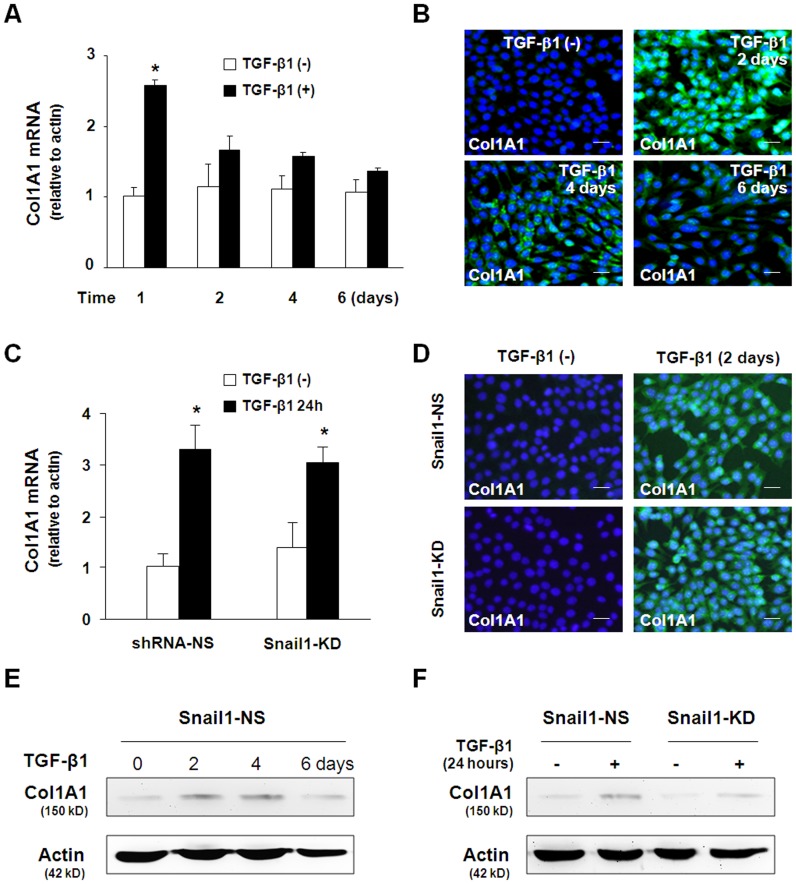
TGF-β1-induced Col1A1 expression is independent of Snail1 up-regulation. TGF-β1 (3 ng/ml) treatment transiently increased Col1A1 mRNA and protein in 603B cells, as determined by qRT-PCR (A and C), immunofluorescent staining (B and D) and Western blot (E and F). Snail1 knockdown did not affect TGF-β1-induced up-regulation of Col1A1 in 603B cells (C–F). Cells stably expressing the Snail1 shRNA or control shRNA were exposed to TGF-β1 (3 ng/ml) for 24 h (for qRT-PCR of Col1A1 in C) and for 2 days (for Conl1A1 protein staining in D and Western blot in E and F). The qRT-PCR results shown represent an average of three independent experiments. Values are means ± SE. *p<0.05 compared to non-TGF-β1-treated cells; shRNA-NS = non specific shRNA control; Snail1-KD = snail shRNA knockdown; Bar = 10 µM.

### Quantitative Reverse-transcription PCR (qRT-PCR)

Total RNA was extracted from 603B cells with TRIzol (Invitrogen). qRT-PCR was performed with ABI-Prism 7900HT (Applied Biosystems) with the SYBR Green polymerase chain reaction master mix (Applied Biosystems). The PCR primers used were as follows: mouse E-cadherin, forward (5′-GGATAGAGAAGCCATTGCCAAGTA-3′) and reverse (5′-TCAAAGACCGGCTGGGTAAA-3′); α-smooth muscle actin (α-SMA), forward (5′-ATCATCACCAACTGGGACGAC-3′) and reverse (5′-TTTCTCCCGGTTGGCCTTAG-3′); Fibronectin1 (Fn-1), forward (5′-TGACACTTATGAGCGCCCTAAAGA-3′) and reverse (5′-TCATGGCAGGGATTTGCAAT-3′); Col1A1, forward (5′-GGGTCTAGACATGTTCAGCTTTGTG-3′) and reverse (5′-ACCCTTAGGCCATTGTGTATGC-3′); Bcl-2-associated X protein (Bax), forward (5′-CAGGATGCGTCCACCAAGAA-3′) and reverse (5′-GCAAAGTGAAAGAGGGCAACCA-3′); BH3 interacting domain death agonist (Bid), forward (5′-ACAGCTAGCCGCACAGTTCAT-3′) and reverse (5′-CAGCATGGCGTTGTCGTTCT-3′); Bcl-2-like protein 11 (Bim), forward (5′-AACCGCAAGCTTCCATACGA-3′) and reverse (5′-TCTTCAGCCTCGGGGTAATC-3′); phosphatase and tensin homolog (Pten), forward (5′-GCGTGCAGATAATGACAAGGAGTA-3′) and reverse (5′-CCTCTGGATTTGATGGCTCCTCTA-3′); p53 up-regulated modulator of apoptosis (Puma), forward (5′-CCCAGAAATGGAGCCCAACT-3′) and reverse (5′-CCAGTATGCTACATGGTGCAGAA-3′); beta-actin, forward (5′-TGGTGGGAATGGGTCAGAA-3′) and reverse (5′-TCTCCATGTCGTCCCAGTTG-3′); Snail1, forward (5′-TAGGTCGCTCTGGCCAACAT-3′) and reverse (5′-TAGGGCTGCTGGAAGGTGAA-3′). Data analysis was performed with ABI-Prism 7900HT SDS2.0 software (Applied Biosystems). Experiments were performed in triplicate and the values were normalized to beta-actin. The cycle threshold (Ct) values were analyzed using the comparative Ct (ΔΔCt) method and the amount of target was obtained by normalizing to the endogenous reference (beta-actin) and relative to the control (non-treated cells).

**Figure 4:**
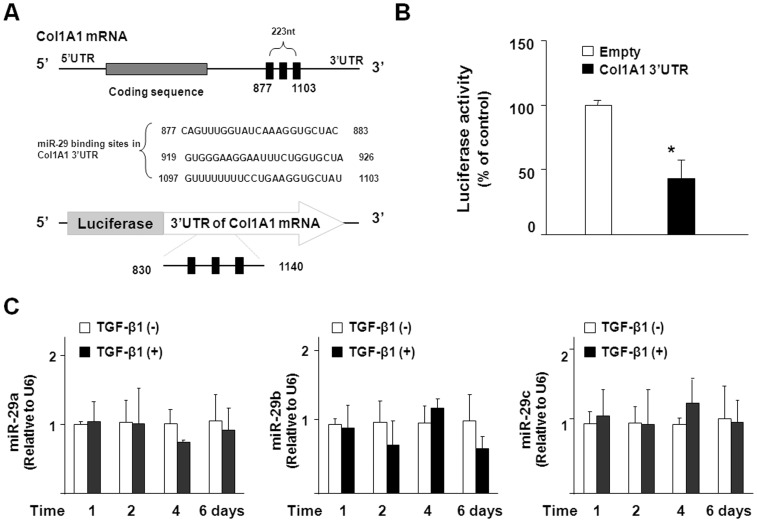
TGF-β1-induced Col1A1 expression is independent of miR-29 downregulation. (A) The schematic of Col1A1 mRNA showed three potential binding sites in its 3′UTR for miR-29 targeting. (B) Col1A1 3'UTR fragments containing miR-29 potential binding sites results in translational suppression in 603B cells as assessed by luciferase reporter assay. The Col1A1 3'UTR sequence covering the potential binding sites for miR-29 was inserted into the pMIR-REPORT luciferase plasmid. The empty pMIR-REPORT luciferase plasmid was used as the control. 603B cells were transfected with the constructs and luciferase analysis was performed 48 h later. Values are means ± SE. *p<0.05 compared to cells transfected with the empty pMIR-REPORT luciferase vector. (C) qRT-PCR analysis revealed no significant down-regulation of miR-29 family members in 603B cells following TGF-β1 treatment for up to 6 days. Data represent an average of three independent experiments.

For analysis of miR-29, total RNA was isolated from cells with the TRIzol reagent (Invitrogen). An amount of 0.05 µg total RNAs was reverse-transcribed by using the Taqman MicroRNA Reverse Transcription Kit (Applied Biosystems). Specific primers and probes for mature miR-29 a/b/c and snRNA RNU6B were obtained from Applied Biosystems. All reactions were run in triplicate. The amount of miR-29a/b/c was obtained by normalizing to snRNA RNU6B and relative to the control (untreated cells). Comparative qRT-PCR was performed in triplicate using Taqman Universal PCR Master Mix (Applied Biosystems) on the Applied Biosystems 7500 FAST real-time PCR System. Relative expression was calculated by using the comparative CT method.

**Figure 5:**
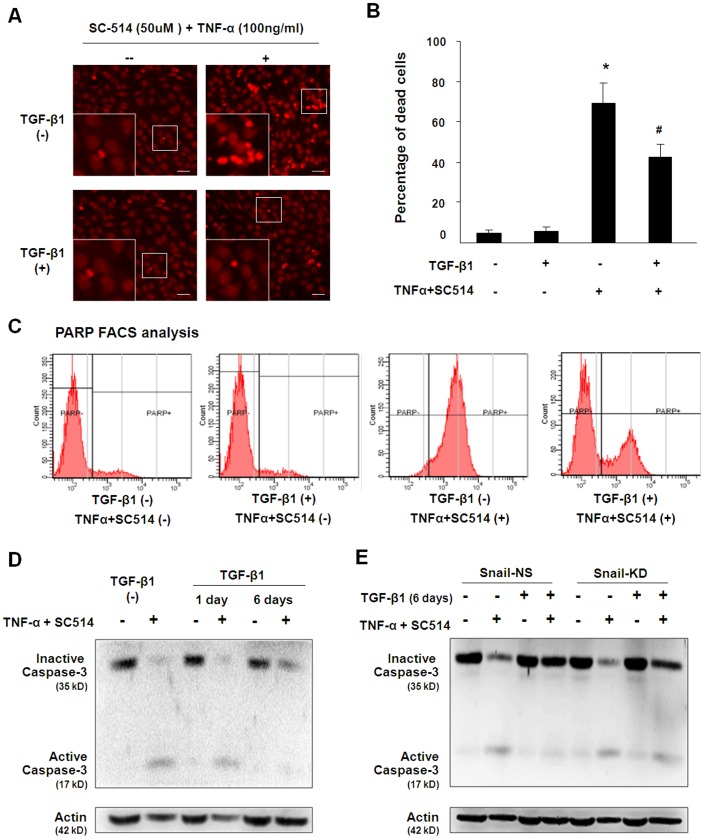
Snail1 is required for TGF-β1-induced apoptosis resistance in 603B cells. (A) TGF-β1 pretreatment inhibits TNF-α- and SC-514-induced cell death. Cells were culture in the absence or presence of TGF-β1 for 5 days and then treated with TNF-α plus SC-514 for additional 24 h. Apoptosis-associated nuclear decomposition in TGF-β1-pretrated or non-pretreated 603B cells as assessed by PI staining. Insets are higher magnifications of the boxed region. Bar = 10 µM. (B) Quantification of frequency of TNF-α/SC-514-induced cell death by trypan blue. Data are presented as percentage of total cell numbers. (C) The PARP cleavage assay for assessing apoptosis by flow cytometry. 603B cells were treated as described above. Both free-floating and attached cells were collected and stained with FITC-conjugated antibody against cleaved PARP followed by FACS analysis. (D) TGF-β1 pretreatment inhibits TNF-α- and SC-514-induced cleavage of caspase-3 in 603B cells. 603B cells were exposed to TGF-β1 for 1 and 5 days, followed by TNF-α plus SC-514 treatment for additional 24 h. The activation of caspase-3 was assessed by Western blot using antibody recognizing both the full-length caspase-3 and cleaved caspase-3 forms. (E) Snai1 is required for the inhibitory effects of TGF-β1 pretreatment on TNF-α- and SC-514-induced cleavage of caspase-3 in 603B cells. 603B stably cells expressing control shRNA or Snail1 shRNA were stimulated with TGF-β1 for 5 days, followed by TNF-α plus SC-514 treatment for 24 h. The activation of caspase-3 was assessed by Western blot. Representative blots in D and E are from three independent experiments and actin was blotted to ensure equal loading. Values are means ± SE. *p<0.05 compared to non- TNF-α/SC514 treated cells; ^#^ *p*<0.05 compared to non-TGF-β1 pretreated cells. shRNA-NS = non specific shRNA control; Snail1-KD = snail shRNA knockdown.

### Western Blot

Whole cell lysates were obtained with the M-PER Mammalian Protein Extraction reagent (Pierce) plus protease inhibitors (1 mmol/L phenylmethanesulfonylfluoride, 10 µg/mL leupeptin, and 2 µg/mL pepstatin). Antibodies to actin, caspase-3, E-cadherin (Sigma-Aldrich), N-cadherin (Cell Signaling), fibroblast-specific protein-1 (FSP-1), vimentin and Col1A1 (Abcam) were used.

**Figure 6:**
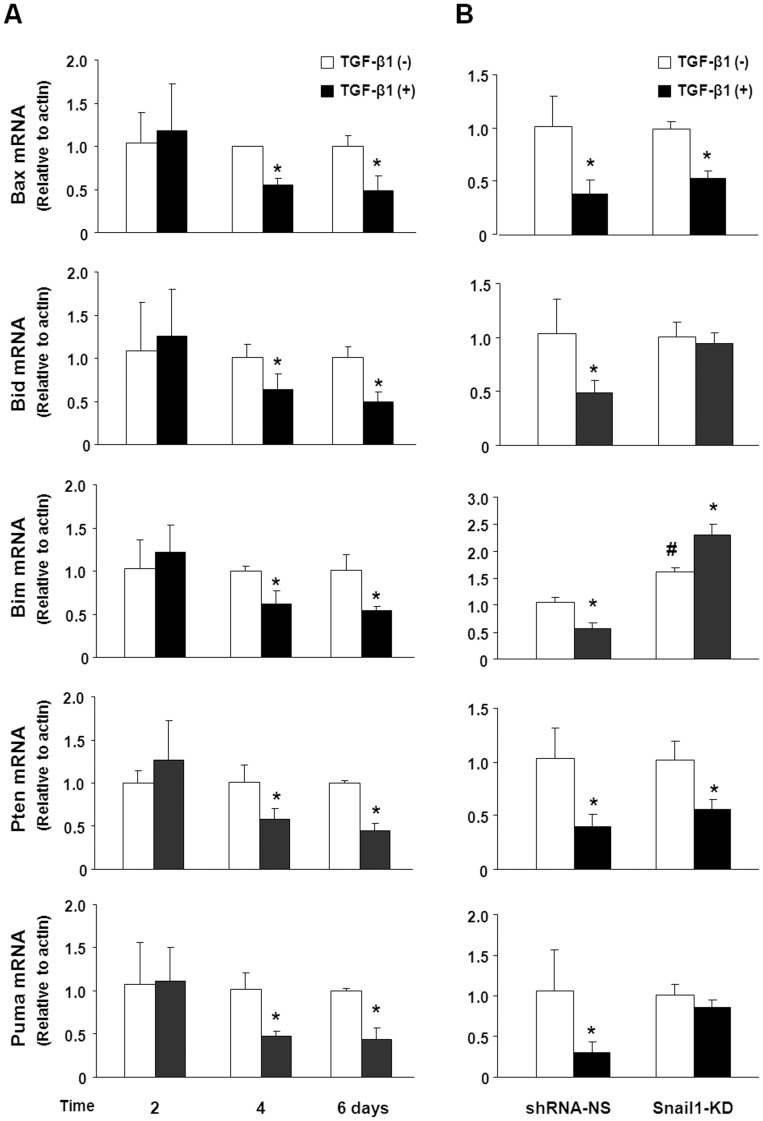
Snail1 contributes to the down-regulation of pro-apoptotic genes induced by TGF-β1. (A) 603B cells were treated with TGF-β1 for indicated periods of time. Alterations of mRNA expression for pro-apoptotic genes Pten, Bim, Bax, Bid and Puma were assessed by qRT-PCR. (B) 603B cells stably expressing control shRNA or Snail1 shRNA were treated with TGF-β1 for 6 days. Changes of pro-apoptotic gene mRNA levels were determined by qRT-PCR. Data represent an average of three independent experiments. Values are means ± SE. *p<0.05 compared to non-TGF-β1 treated cells; ^#^ *p*<0.05 compared to shRNA-NS control. shRNA-NS = non specific shRNA control; Snail1-KD = snail shRNA knockdown.

### Luciferase Reporter Constructs and Luciferase Assay

A 280 bp fragment from Col1A1 3′UTR containing three potential miR-29 binding sites was cloned into the multiple-cloning site of the pMIR-REPORT Luciferase vector (Ambion). The empty pMIR-REPORT vector was used as a control. We then transfected cultured cells with each reporter construct. Luciferase activity was measured 24 h later and normalized to the control β-galactosidase level.

### Cell Death and Propidium Iodide (PI) Staining Assay

For trypan blue staining, 5000 cells were plated in each well of a 6-well cluster dish and TGF-β1 was added at indicated time points. Medium was then removed; TNF-α (100 ng/ml) and SC-514 (50 nM) were added to induce apoptosis for 24 h. Floating cells were collected by centrifuge at 200 g for 5 min. The attached cells were trypsinized and combined with floating cells for staining. The numbers of dead and viable cells were counted under the microscope. For PI staining, 5000 cells were plated in 6 cm dishes and treated as described above. Cells were washed with phosphate buffered saline (PBS) and then fixed for 15 min with 4% formaldehyde in PBS. Cells were then washed with PBS and stained with PI for 5 min. Dye was removed, cell monolayers were washed with PBS, and apoptosis was assessed under the fluorescence microscope.

### Immunofluorescence (IF) Staining

The 603B cells for immunofluorescence staining of E-cadherin and Col1A1 were plated in 8-well culture slides and treated in the same method as above described. Cells were washed with PBS three times and fixed with 4% paraformaldehyde in PBS for 10 min at room temperature and permeabilized in 0.5% Triton X-100/PBS for 5 min at room temperature. After washing with PBS for 5 min (three times), cells were incubated with blocking buffer (Cell Signaling IF protocol) containing 5% serum (from same species as secondary antibody) for 1 h at room temperature, and then incubated with the specific primary antibodies. The following primary antibodies were used for immunostaining: rat anti-E-cadherin (clone ECCD-2) (Sigma-Aldrich), and rabbit anti-procollagen/type I collagen (Chemicon, Temecula, CA). Alexa fluor-488 conjugated secondary antibodies were used. Cells were co-stained with 4′,6-diamidino-2-phenylindole (DAPI), to visualize the nuclei. Stained cells were mounted with fluorescent mounting medium (Dako Cytomation), and assessed by a conventional fluorescent microscopy (Olympus BX51/DP71). The exposure time for FITC and DAPI signals was 2 seconds and 0.08 seconds, respectively.

### The Enzyme Poly (ADP-ribose) Polymerase (PARP) Cleavage Assay for Assessing Apoptosis by Flow Cytometry

The PARP cleavage was determined using FITC-conjugated anti-PARP (85-kDa fragment) antibody (Abcam) to quantify apoptotic cells, as previously reported [29]. Cells were fixed with 0.01% formaldehyde for 15 min, and were then incubated with 200 µl of permeabilizing solution (0.5% BSA, 0.02% NaN3, and 0.5% Tween 20, in PBS) for 30 min at 37°C. After permeabilization, cells were stained with the anti-PARP antibody conjugated with FITC for 1 h. Cells were washed with PBS and analyzed by flow cytometry [29].

### Data Analysis

All values are given as mean ± SE. Means of groups were compared with the Student’s *t* test (unpaired) or ANOVA test when appropriate. *p* values <0.05 were considered statistically significant.

## Results

### TGF-β1 Induces EMT-like Phenotypic Changes in 603B Cells

Murine immortalized 603B cholangiocytes were seeded at low density and cultured in the medium with or without TGF-β1 (3 ng/ml) for up to 6 days. In the absence of TGF-β1, 603B cells maintained epithelial morphology during the culture period. In the presence of TGF-β1, the cells gradually displayed morphological appearances of mesenchymal cells and after 6 days of TGF-β1 treatment, the majority of cells became spindle-shaped (Fig. 1*A*). These morphological features suggested that TGF-β1 may induce EMT-like phenotypic changes in 603B cells.

EMT is characterized by loss of epithelial cell polarity, loss of cell-cell contacts, and acquisition of mesenchymal markers and phenotypic traits that include increased cell motility. E-cadherin is universally expressed by epithelial cells and plays essential roles to form the tight junctions between epithelial cells. Loss of E-cadherin expression is a marker for the occurrence of EMT. We found that after 2 days of TGF-β1 treatment, E-cadherin mRNA levels were steadily decreased in 603B cells as compared with the controls (p<0.05, Fig. 1*B*). TGF-β1 treatment did not significantly affect actin mRNA level during the culture (data not shown) and thus, it was used as the internal control to normalize the qRT-PCR data. Western blot analysis revealed a similar but delayed decrease in E-cadherin protein level in TGF-β1-treated cells, comparing with mRNA changes. Specifically, TGF-β1 treatment for 4 days only induced a modest decrease of E-cadherin protein in 603B cells, and a dramatic decrease of E-cadherin protein was detected 6 days after TGF-β1 stimulation (Fig. 1*C*). Decreased expression of E-cadherin in TGF-β1-treated 603B cells was further confirmed by immunofluorescent staining. As shown in Fig. 1*D*, whereas cells cultured in the absence of TGF-β1 predominantly expressed E-cadherin on the cell membrane, cells in the presence of TGF-β1 gradually lost E-cadherin expression. In contrast, TGF-β1-treated cells showed an increased expression of N-cadherin, a mesenchymal marker upregulated during EMT [20]. Expression of N-cadherin at both the message and protein levels was increased in cells in response to TGF-β1 treatment as assessed by qRT-PCR (Fig. 1*B*) and Western blot (Fig. 1*C*). In addition, cells undergoing EMT frequently express higher levels of α-SMA, Fn-1, FSP-1 and vimentin [20]. Nevertheless, no changes in α-SMA and Fn-1 mRNA levels were detected in cells following TGF-β1 treatment by real-time PCR (Fig. 1*E*). TGF-β1 stimulation failed to induce the expression of FSP-1 and vimentin at the protein levels (Fig. 1*F*). These data together suggested that TGF-β1 alone may only induce a partial EMT in 603B cells.

### Snail1 is Required for TGF-β1-induced EMT-like Changes in 603B Cells

Snail1 is an important downstream effector of TGF-β1 signaling to induce EMT in various cells and functions as a transcriptional suppressor of E-cadherin [20]. We examined whether TGF-β1 influences Snail1 expression in 603B cells. Consistent with data from previous reports [27], [17], we found that Snail1 mRNA levels were rapidly increased after TGF-β1 stimulation (Fig. 2*A*) and remained a modest increase (1.3–1.5 folds) at 2, 4 and 6 days after treatment, indicating that the induction of Snail1 may be an early event of TGF-β1-induced EMT-like changes.

To elucidate whether up-regulation of Snail1 contributed to the EMT-like changes, stable Snail1 knockdown in 603B cells was achieved by a specific shRNA to Snail1. As expected, Snail1 shRNA significantly reduced the basal Snail1 mRNA (Fig. 2*B*) and protein (Fig. 2*C*) levels, compared with the control shRNA. Moreover, TGF-β1-induced up-regulation of Snail1 was abolished in cells stably expressing Snail1 shRNA (Fig. 2*B and 2C*). We then cultured these cells in the absence or presence of TGF-β1 for 6 days and examined the morphological changes and expression patterns of E-cadherin and N-cadherin. As shown in Fig. 2*D*, Snail1 knockdown attenuated TGF-β1-induced EMT-like morphological alterations. Snail1 knockdown did not significantly change the basal expression of E-cadherin and N-cadherin at both the message and protein levels (Fig. 2*E and 2F*). However, TGF-β1-induced downregulation of E-cadherin was significantly impaired in the Snail1 knockdown cells. Moreover, Snail1 knockdown attenuated the upregulation of N-cadherin mRNA induced by TGF-β1 (Fig. 2*E*). Western blot analysis showed that Snail1 knockdown blocked TGF-β1-induced downregulation of E-cadherin and upregulation of N-cadherin in 603B cells (Fig. 2*F*). Taken together, these results clearly indicate that Snail1 was induced by TGF-β1 in 603B cells and this induction was required for TGF-β1-induced EMT-like changes.

### Snail1 is Dispensable for TGF-β1-induced Col1A1 Expression in 603B Cells

It has been proposed previously that epithelial cells undergoing EMT may be an important source of ECM protein-producing cells during tissue fibrosis. Therefore, we examined whether TGF-β1 could induce the expression of Col1A1, a gene encoding collagen α1(I) which is predominantly accumulated in the portal region during liver fibrosis. Col1A1 mRNA levels were increased 1 day after TGF-β1 exposure but decreased to the basal level at 2 days after TGF-β1 stimulation (Fig. 3*A*). Immunofluorescent staining and Western blot showed similar but delayed changes in Col1A1 protein levels in TGF-β1-treated cells (Fig. 3*B and 3E*). To test the role for snail1 in TGF-β1-induced up-regulation of Col1A1, we evaluated the Col1A1 mRNA levels in Snail1 knockdown 603B cells cultured in the absence or presence of TGF-β1 for 24 h (for Col1A1 mRNA) and 48 h (for Col1A1 protein). As shown in Fig. *3D and 3F*, Snail1 knockdown neither affected the basal level of Col1A1 nor prevented TGF-β1-induced Col1A1 expression in 603B cells. These data suggested that although Col1A1 expression is upregulated by TGF-β1 signaling in cultured 603B cells, production of Col1A1 may be transient and not dependent on snail1-associated EMT-like phenotypic alterations.

### miR-29s are not Involved in TGF-β1-induced Col1A1 Expression in 603B Cells

Col1A1 is a target for miR-29 [24]. It has recently been shown that decreased miR-29 expression results in the excessive production of Col1A1 in cardiac fibrosis and liver fibrosis patients [23], [24]. To explore the potential involvement of miR-29 in TGF-β1-induced Col1A1 upregulation in 603B cells, we generated a luciferase vector which contains a 280-bp fragment of Col1A1 3′UTR with three conserved seed sequences for miR-29 targeting (Fig. 4*A*), conserved for miR-29a, -29b, and -29c [24]. As shown in Fig. 4*B*, the luciferase activity in 603B cells transfected with this construct was much lower than that in cells transfected with the empty control plasmid, suggesting repression of Col1A1 translation by endogenous miR-29s. Nevertheless, qRT-PCR analysis showed no significant change for the expression of miR-29 family members after TGF-β1 exposure (Fig. 4*C*). Thus, although Col1A1 is a target for miR-29, miR-29-mediated posttranscriptional mechanisms may not be involved in TGF-β1-induced Col1A1 expression in 603B cells.

### TGF-β1 Induces Apoptotic Resistance in 603B Cells in a Snail1-dependent Manner

Cholangiocytes are aberrantly accumulated during liver fibrosis, suggesting the impaired balance between cell growth and cell death. Interestingly, during the liver development, high level TGF-β1 favors the accumulation of ductular-like cells [30]. We then evaluated whether TGF-β1 treatment promotes 603B cells survival *in vitro*. Cells were treated with TNF-α plus the NF-κB inhibitor SC-514 for 24 h. This treatment resulted in substantial cells death in 603B cells (Fig. 5*A and 5B*). PI staining revealed apoptotic nuclear fragmentation in a considerable portion of these cells after TNF-α and SC-514 treatment, suggesting occurrence of apoptosis (Fig. 5*A*). Pre-treatment with TGF-β1 for 5 days exerted a significant protective effect against TNF-α/SC-514-induced cell death (Fig. 5*A and 5B*). Consistent with these data, PARP cleavage assay by FACS showed that TGF-β1 pretreatment decreased apoptotic cell death induced by TNF-α/SC-514 treatment in 603B cells (Fig. 5*C*). To further demonstrate the nature of TNF-α/SC-514-induced cells death, we evaluated the caspase-3 activation by Western blot. Without TNF-α/SC-514 stimulation, 603B cells predominantly expressed the uncleaved procaspase-3. Addition of TNF-α plus SC-514 switched inactive procaspase-3 into active (cleaved) caspase-3, substantiating the induction of apoptosis (Fig. 5*D*). 603B cells after 5 days of TGF-β1 exposure showed no significant difference in the expression level of inactive procaspase-3 as compared to non-treated cells. However, TNF-α/SC-514 induced caspase-3 cleavage was significantly attenuated (Fig. 5*D*). Interestingly, pre-treatment with TGF-β1 for 1 day failed to prevent TNF-α/SC-514 induced caspase-3 activation (Fig. 5*D*), suggesting the acquisition of apoptotic resistance by TGF-β1 might be a relatively late event.

To explore the underlying mechanisms, we measured associated caspase-3 activation in snail1 knockdown 603B cells. Cells stably expressing control shRNA or Snail1 shRNA were treated with TGF-β1 for 5 days and then exposed to TNF-α plus SC-514 for 24 h. Western blot analysis showed that TGF-β1 treatment significantly decreased caspase-3 activation in cells transfected with the control shRNA. In contrast, the suppressive effect of TGF-β1 treatment on TNF-α/SC-514 induced caspase-3 activation was attenuated in the snail1 knockdown cells (Fig. 5*E*), suggesting that Snail1 might contribute to TGF-β1-induced apoptotic resistance in 603B cells.

Snail1 functions as a transcriptional factor to suppress the expression of multiple genes, including several pro-apoptotic genes, Bax, Bid, Bim, Pten and Puma [27], [31]–[33]. We found that the mRNA levels of these genes were steadily down-regulated in cells 4 days and 6 days after TGF-β1 treatment (Fig. 6*A*). Snail1 knockdown attenuated or even reversed TGF-β1 suppressive effects on some of these genes. Specially, Snail1 knockdown increased the basal expression of Bim in 603B cells, which was further induced by TGF-β1 treatment. Although Snail1 knockdown did not enhance basal Bid and Puma expression, downregulation of their expression induced by TGF-β1 was attenuated in the Snail1 knockdown cells (Fig. 6*B*). No significant impact on expression of Bax and Pten was detected in the Snail1 knockdown cells. These data suggest that Snail1 may be a key mediator for TGF-β1 pathway to modulate cell survival by regulating pro-apoptotic genes.

## Discussion

Whether cholangiocytes can transdifferentiate into myofibroblast-like cells via EMT to promote liver fibrosis development is still under debate [34]. Cholangiocytes display morphological features of EMT during hepatic fibrosis [4], [7]–[9]. However, recent data from cell fate mapping experiments suggested that there were no FSP1 or α-SMA positive cells derived from cholangiocytes during murine liver fibrosis, suggesting that cholangiocytes might not be a key origin of myofibroblast during liver fibrosis [35]–[37]. In this study, we examined EMT-associated reactions in cholangiocytes in response to TGF- β1 *in vitro*, using a non-tumorigenic mouse 603B cholangiocyte cell line. Consistent with the results from cell fate mapping experiments *in vivo*, we found that TGF-β1 did not change the expression of FSP1 or vimentin in 603B cells. However, TGF-β1 induced EMT-like phenotypic alterations, promoted Col1A1 production and increased apoptotic resistance in 603B cells. Interestingly, TGF-β1-induced Col1A1 production appears to be independent of EMT-like alternations. These data suggest that TGF-β1 induces EMT-like differentiation in 603B cells. However, EMT-like differentiation of cholangiocytes may not be required for collagen production; rather, it promotes cell survival capacity.

Previous studies indicate that TGF-β1 is significantly elevated during liver fibrosis and plays a pivotal role in the development of liver fibrosis [16]. Our results showed that 603B clearly lost the epithelial morphological features after TGF-β1 treatment. Specifically, these cholangiocytes underwent morphological changes to become spindle-shaped cells in response to continuous TGF-β1 stimulation, which might reflect cholangiocyte shape changes observed during liver fibrosis [18]. More importantly, treated cells also gradually lost the expression of E-cadherin, an important protein to mediate the formation of tight junctions between epithelial cells [20]. TGF-β1 treatment also significantly increased N-cadherin expression in 603B cells, suggesting EMT-like differentiation. Of note, TGF-β1 stimulated 603B cells to enhance expression of Snail1, which is mechanically a critical downstream mediator for TGF-β1-induced EMT [15]. Consistently, we found that downregulation of E-cadherin, upregulation of N-cadherin and morphological alterations were largely dependent on the Snail1 signaling.

Liver fibrosis is characterized by the accumulation of excessive amounts of ECM proteins, which leads to the destruction of hepatic architecture. There is increasing interest in investigating whether epithelial cells undergoing EMT directly contribute to the ECM deposition, because uncovering the unique EMT signaling molecules associated with collagen production might help to identify novel therapeutic targets. Data from this study indicate that development of EMT is not a prerequisite for collagen production in cholangiocytes in response to TGF-β1 stimulation *in vitro*. Consistent with results from previous studies [17], [19], [35], [36], we found that 603B cells increased Col1A1 production in response to TGF-β1 stimulation. However, Col1A1 expression was only transiently induced by TGF-β1 stimulation, and not paralleled with the persistent upregulation of N-cadherin or downregulation of E-cadherin in 603B cells undergoing EMT-like alterations. Moreover, upregulation of Col1A1 appears to be independence of Snail1 signaling. In fact, although most TGF-β1-induced EMT-like alterations were abolished in Snial-1 knockdown cells, these cells were still responsive to TGF-β1 to increase Col1A1 expression. It’s possible that TGF-β1 might activate distinct pathways to induce Col1A1 production and EMT-like changes in cholangiocytes. Similar to our results, TGF-β1-induced Col1A1 production in mouse hepatocytes did not parallel the occurrence of EMT [36].

Recent studies demonstrated that miRNAs, such as miR-29 family members, might play a role in the control of cardiac and liver fibrosis [23], [24]. It has experimentally been confirmed that miR-29s can target the 3′UTRs of several collagen mRNAs and suppress the expression of multiple collagens including Col1A1 [24]. Down-regulation of miR-29s has been reported in fibrotic areas of cardiac and liver fibrosis. The precise molecular mechanisms underlying miR-29 dysregulation during fibrosis are unknown. TGF-β1 has been demonstrated to suppress miR-29 expression in the cardiac fibroblast and HSC cells *in vitro* [23], [24]. We found that the luciferase activity in 603B cells transfected with the construct covering the miR-29 binding sites within the 3′UTR of Col1A1 was significant suppressed, indicating that endogenous miR-29 might help to prevent excessive expression of Col1A1. Nevertheless, expression of miR-29a/b/c in 603B cells was unaffected in response to TGF-β1 stimulation. Therefore, TGF-β1 may regulate miR-29 expression in a cell type-specific manner. Moreover, TGF-β1-induced Col1A1 expression in 603B cells *in vitro* is independent of miR-29 expression.

Cholangiocyte accumulation is a poor predictor for hepatic fibrosis. Our study demonstrated that TGF-β1-treated 603B cells were more resistant to TNF-α/SC-514-induced apoptosis. Snail1 knockdown significantly attenuated the protective effects of TGF-β1 on 603B cells, suggesting that TGF-β1-induced EMT-like alterations might contribute to cholangiocyte accumulation during liver fibrosis via suppressing apoptotic cell death. Increasing evidence supports that induction of EMT is generally associated with reduced apoptosis and the molecular mechanisms are not fully understood. We found a modest downregulation of multiple pro-apoptotic genes, including Bax, Bid, Bim, Pten, and Puma, in 603B cells after continuous TGF-β1 stimulation. Snail1 knockdown abolished down-regulation of Bid, Bim and Puma induced by TGF-β1 exposure, further supporting the involvement EMT-associated signaling in the development of apoptotic resistance in TGF-β1-treated cells. Genome-wide ChIP analysis suggested that Snail1 can bind the promoters of many apoptosis-associated genes when it was overexpressed in ovarian cancer cells [34]. Whether TGF-β1-induced Snail1 can directly silence these pro-apoptotic genes merits further investigation. In addition, the regulatory effects of Snail1 on apoptosis might not limit to silencing pro-apoptotic genes. A recent study suggested that snail1 knockdown prevented the upregulation of anti-apoptotic molecules, Bcl-xL and Mcl-1, in mouse hepatocytes induced by TGF-β1 [27]. Obviously, many anti-apoptotic and pro-apoptotic molecules and activation of multiple EMT-associated signal pathways are involved. Targeting accumulated proliferative cholangiocytes has been shown to attenuate liver fibrosis in mice [38]. A comprehensive evaluation of the role for EMT signaling in cholangiocyte apoptotic resistance during liver fibrosis may help to identify new targets for therapeutic intervention. It will be also of interest to extend these studies to other cholangiocyte cell lines, as well as to determine the role of TGF-β1-induced cholangiocyte EMT in ECM production during liver fibrosis *in vivo.*
